# Evaluation of Carotid Artery Atherosclerosis and Arterial Stiffness in Cardiovascular Disease Risk: An Ongoing Prospective Study From the Kailuan Cohort

**DOI:** 10.3389/fcvm.2022.812652

**Published:** 2022-05-02

**Authors:** Wen Li, Yan Wang, Shuohua Chen, Jianqiu Zhao, Qi Su, Yanfeng Fan, Shouling Wu, Jun Li, Jiang Hong

**Affiliations:** ^1^Department of Ultrasound, Shanghai Jiao Tong University Affiliated 6th People's Hospital, Shanghai Institute of Ultrasound in Medicine, Shanghai, China; ^2^Department of Cardiology, Kailuan General Hospital Affiliated to North China University of Science and Technology, Tangshan, China; ^3^Department of Anesthesiology, Anting Hospital, Shanghai, China; ^4^Department of Internal and Emergency Medicine, Shanghai General Hospital, Shanghai, China; ^5^Department of Cardiology, Shanghai General Hospital, Shanghai, China

**Keywords:** atherosclerosis, arterial stiffness (AS), carotid plaque (CP), cardiovascular disease, all-cause mortality

## Abstract

**Objective:**

To assess whether carotid artery ultrasonography and brachial-ankle pulse wave velocity (baPWV) measurement can accurately predict cardiovascular and cerebrovascular events, and all-cause mortality in patients with cardiovascular diseases (CVD).

**Methods:**

Patients from the Kailuan Study Stroke Cohort (Tangshan, China) who underwent carotid artery ultrasonography and baPWV measurement between June 2010 and June 2011 were included in this study. The effects of carotid plaque, baPWV, and their combination on cardiovascular events, including myocardial infarction (MI), cerebral ischemic stroke, cerebrovascular events, and all-cause mortality, were evaluated using Kaplan-Meier analysis and Cox proportional hazards regression.

**Results:**

A total of 4,899 participants (59.7% males; 54.18 ± 11.52 years old) were analyzed. During a mean follow-up of 5.68 ± 0.66 years, the incidence of cardiovascular events and all-cause mortality were 4.94‰ person-years and 7.02‰ person-years, respectively; 32.8% of participants had both carotid artery atherosclerosis and increased arterial stiffness. A high baPWV alone was associated with an increased risk of CVD events [hazard ratio (HR): 2.68; 95% confidence interval (95% CI): 1.20–6.00; *P* = 0.007] and cerebral infarction (HR: 5.92; 95% CI: 1.76–19.93; *P* = 0.004), but not with MI or all-cause death. The presence of both carotid plaque and high baPWV was highly associated with an increased risk of CVD events (HR: 4.65; 95% CI: 2.06–10.45; *P* < 0.001) and cerebral infarction (HR: 9.21; 95% CI: 2.71–31.19; *P* < 0.001), but not with MI or all-cause death. Similar results were obtained by the Kaplan-Meier analyses.

**Conclusion:**

The presence of carotid plaque and high baPWV were associated with a high risk of CVD events and ischemic stroke. Moreover, the combination of carotid artery ultrasonography and baPWV measurement could predict the risk for CVD ability more accurately than a single measurement alone.

## What Is Already Known

1. Higher arterial stiffness is a risk factor for cardiovascular disease, which can predict adverse cardiovascular outcomes
2. Carotid artery atherosclerosis detected by ultrasound is an important risk factor for cardiovascular disease

## What the Present Study Adds

The presence of carotid plaque and high baPWV is associated with a high risk of CVD events and ischemic stroke. Moreover, the combination of carotid artery ultrasonography and baPWV measurement could predict the risk for CVD ability more accurately than a single measurement alone.

## Introduction

Cardiovascular disease (CVD) is the leading cause of mortality worldwide. It has been estimated that some 18 million people died from CVD in 2019, representing 32% of all global deaths; among those, 85% were due to stroke and heart attack (1). Older age, smoking, family history of CVD, hypertension, diabetes mellitus (DM), obesity, increased levels of low-density lipoprotein-cholesterol (LDL-C) and triglycerides, and decreased levels of high-density lipoprotein-cholesterol (HDL-C) are the major risk factors associated with CVD (2). Risk stratification for CVD can be used to evaluate a patient's risk of developing CVD, thus reducing morbidity and prolonging survival.

Atherosclerosis is the pathologic basis of CVD. In its early stages, atherosclerosis decreases arterial elasticity and compliance and increases arterial stiffness, which results from damage to the elastic fibers and increased collagen deposition in the vessel wall. The increase in arterial stiffness occurs before any obvious changes in vascular wall structure or the appearance of clinical symptoms or signs (3). Furthermore, arterial stiffness is considered an important risk factor for CVD that may predict adverse cardiovascular outcomes (4–8). Therefore, evaluating arterial stiffness might be useful for identifying an early-stage vascular disease.

Advanced atherosclerosis with plaque formation in the intimal layer of cerebral blood vessels has a major role in the pathogenesis of ischemic stroke, which is an important cause of death and disability in adults (9, 10). The incidence of stroke has been increasing in China in recent years (11, 12). Ischemic stroke accounts for 60–80% of all strokes (10), and stenosis or obstruction of the carotid arteries by atherosclerotic plaques is a major cause of ischemic stroke (13).

Pulse wave velocity (PWV) is a gold-standard indicator for evaluating arterial stiffness (14). Brachial-ankle PWV (baPWV) (15, 16) is a convenient measurement that has been widely applied for the assessment of vascular risk in the general population in Asia (17, 18). On the other hand, carotid atherosclerosis detected by ultrasound is an important risk factor for CVD (19–26). Although previous research suggested that carotid plaques and baPWV are risk factors for CVD, few studies have examined whether the risk of CVD is higher in the presence of both factors.

The Kailuan study is an ongoing community-based study aiming to evaluate the risk factors and interventions for cardiovascular, cerebrovascular, and related diseases. Some of the participants in the Kailuan study underwent both carotid ultrasonography and baPWV measurement, thus providing us with an opportunity to assess whether carotid artery ultrasonography and baPWV measurement can accurately predict cardiovascular and cerebrovascular events, and all-cause mortality in patients with CVD.

## Materials and Methods

### Study Design and Population

Patients from the Kailuan Study Stroke Cohort (Tangshan, China) (27) who underwent carotid artery ultrasonography and baPWV measurement between June 2010 and June 2011 were included in this study. The Kailuan study is an ongoing prospective study initiated in 2006 by Kailuan (Group) Co., Ltd. In Tangshan City, a large littoral metropolis located 180 km southeast of Beijing. The study has been conducted in accordance with the guidelines of the Helsinki Declaration and was approved by the ethics committees of Kailuan General Hospital (Tangshan, China) and Beijing Tiantan Hospital (Beijing, China). All participants provided informed written consent. This study was registered at the China Clinical Trial Registry (registration number: ChiCTR-TNC-11001489).

A total of 5,440 participants in the stroke cohort of the Kailuan study who underwent physical examinations between June 2010 and June 2011 were selected using stratified random sampling. The inclusion criteria were: (1) age ≥ 40 years; (2) no history of stroke (excluding lacunar infarction), transient ischemic attack, or coronary artery disease at baseline as assessed using a validated questionnaire and by experienced physicians; (3) those who underwent bilateral carotid ultrasonography and baPWV measurement. The exclusion criteria were: (1) serious physical disability; (2) inability to undergo physical check-ups/examination; (3) data required for the analysis were missing.

### Clinical Evaluation and Definitions

All participants underwent a standardized interview and a clinical examination. Demographic and clinical information, including age, sex, smoking status, alcohol consumption, regular medications, and blood pressure (BP), were collected from all patients (27). Fasting blood samples were drawn to measure the concentrations of fasting blood glucose (FBG), HDL-C, LDL-C, triglycerides, and total cholesterol (TC). Smoking was self-reported as “yes” or “no.” Alcohol consumption was defined as an intake of ≥100 mL/day for more than 1 year. Hypertension was defined as a history of hypertension, systolic BP (SBP) ≥ 140 mmHg, diastolic BP (DPB) ≥90 mmHg, or use of antihypertensive medications. DM was diagnosed if the subject had a history of DM, was currently using insulin or oral hypoglycemic agents, or had a fasting blood glucose level ≥126 mg/dL. Hyperlipidemia was defined as a history of hyperlipidemia, total blood cholesterol level ≥220 mg/dL, triglyceride level ≥150 mg/dL, or current use of lipid-lowering drugs.

For some analyses, the participants were divided into subgroups according to age, SBP, DBP, FBG, LDL-C, HDL-C, TC, and body mass index (BMI). The age subgroups were 40–49 and ≥50 years. The SBP subgroups were <130 mmHg (normal), 130–139, 140–159, 160–179, and >180 mmHg. The DBP subgroups were <85 mmHg (normal), 85–89, 90–99, 101–109, and >110 mmHg (28). The FBG subgroups were <6.1 mmol/L (hypoglycemia), 6.1–6.9 mmol/L (normoglycemia), and ≥7.0 mmol/L (hyperglycemia) (28). The LDL-C subgroups were <4.1 mmol/L (normal) and ≥4.1 mmol/L (high). The HDL-C subgroups were <1.0 mmol/L (low) and ≥1.0 mmol/L (normal) (28). The TC subgroups were <6.2 mmol/L (normal) and ≥6.2 mmol/L (high) (28). The BMI subgroups were <24.0 kg/m^2^ (normal weight), 24.0–27.9 kg/m^2^ (overweight), and ≥28 kg/m^2^ (obese) (29).

### baPWV

baPWV was measured using a BP-203 RPE III networked arterial stiffness detection device (Omron Healthcare Co. Ltd, Dalian, China). The participants were placed in a supine position after at least 5 min of rest. The distance between the baPWV sampling points was then calculated automatically according to the subject's height. The path length from the suprasternal notch to the ankle (La) was calculated using the formula: La = 0.8129 × height (cm) + 12.328. The path length from the suprasternal notch to the brachium (Lb) was calculated using the formula: Lb = 0.2195 × height (cm)−2.0734. The baPWV (cm/sec) was calculated as (La–Lb)/Tba, where Tba is the time interval between the front waves of the brachial waveform and ankle waveform during simultaneous measurements of baPWV on both sides of the body (30). The highest baPWV measurement was chosen as the representative value for each individual (31). According to the 2017 ACC/AHA Guideline for the Prevention, Detection, Evaluation, and Management of High Blood Pressure in Adults (32), baPWV <1,400 cm/s was regarded as normal arterial stiffness and baPWV ≥ 1,400 cm/s indicated arteriosclerosis.

### Evaluation of the Carotid Arteries for Atherosclerotic Plaques

All study participants underwent bilateral carotid duplex ultrasound (Philips iU-22 Ultrasound System, Philips Medical Systems, Bothell, WA, US) to evaluate the presence of the carotid plaques. A carotid artery plaque was defined as a focal structure invading the arterial lumen by at least 0.5 mm or 50% of the surrounding intima-media thickness value or demonstrated a thickness > 1.5 mm as measured from the media-adventitia interface to the intima-lumen interface (33).

### Follow-Up and Evaluation of Clinical Outcomes

The participants were followed up by face-to-face interviews. Routine medical examinations were performed every 2 years until December 31, 2016, or the occurrence of the event of interest, or death. Outcome information for patients unable to attend the face-to-face follow-up interviews was obtained by checking the hospital medical records and the patient's medical insurance records. The study endpoints included the first occurrence of a CVD event (stroke or myocardial infarction [MI]) and all-cause mortality. The diagnosis of stroke was done according to World Health Organization criteria (34) and was based on signs, symptoms, neuroimaging data (from computed tomography or magnetic resonance imaging), and other diagnostic reports as detailed previously (35). MI was defined following the 2007 universal definition (36). Deaths from all causes were confirmed by checking death certificates from the provincial vital statistics offices. All outcomes were approved by the Data Safety Monitoring Board and the Arbitration Committee for Clinical Outcomes.

### Statistical Analyses

Baseline characteristics were presented in descriptive statistics, with mean ± SD given for normally distributed (according to the Kolmogorov-Smirnov test) continuous variables or median (interquartile range) given for non-normally distributed continuous variables. Categorical variables were presented as numbers (percentages). Continuous variables were compared between groups using analysis of variance (ANOVA), followed by between-group *post-hoc* analysis using Fisher's least square difference (LSD) if significant differences were revealed. Categorical variables were analyzed using the Chi-squared test. Cox proportional hazards regression was used to estimate the risk of events by calculating the hazard ratios (HRs) and 95% confidence intervals (CIs). The regression models were adjusted for age and sex (model 1) and age, sex, smoking status, alcohol consumption, BMI, FBG, TC, LDL-C, HDL-C, SBP, DBP, antihypertensive drug use, and hypoglycemic drug use (model 2). The rates of outcome events were compared across groups using the Kaplan-Meier method and the log-rank test. The ability of high baPWV to discriminate between CVD and cerebral infarction risk was assessed using C-statistics, and the discrimination ability of the model was assessed by comparing the changes in C-statistics after the addition of carotid plaque. In order to assess the utility of risk classification for CVD and cerebral infarction, we calculated the net reclassification index (NRI) and integrated discrimination improvement (IDI) after adding carotid plaques to high baPWV. All statistical tests were two-tailed; a *P*-value of 0.05 was considered as statistically significant. The analyses were performed using SAS 9.3 (SAS Institute, Cary, NC, USA).

## Results

### Baseline Characteristics of the Study Population

**Figure 1:**
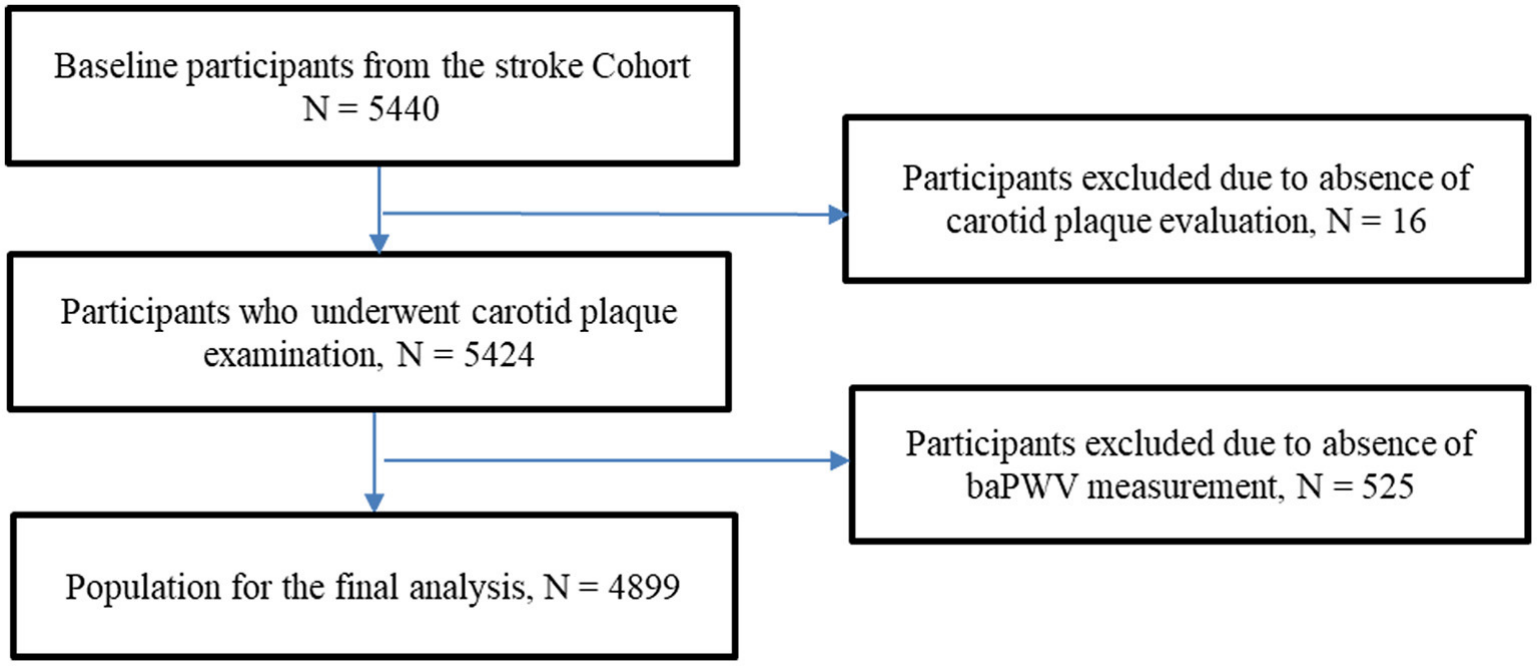
Flow chart showing enrolment of the study participants.

Among 5,440 subjects screened for analysis, 16 were excluded because the baseline carotid plaque data were unavailable, and 525 due to missing data for baseline baPWV. Therefore, 4,899 participants (59.7% males) with a median age of 51.60 (14.08) years were included in the final analysis (Figure 1). The baseline characteristics of the participants are presented in Table 1.

**Table 1:** Baseline characteristics of the study population.

	**All** **participants** **(***n*** = 4,899)**	**No carotid plaque, normal baPWV** **(***n*** = 1,545)**	**Carotid plaque,** **normal baPWV** **(***n*** = 398)**	**No carotid plaque,** **higher baPWV** **(***n*** = 1,348)**	**Carotid plaque,** **higher baPWV** **(***n*** = 1,608)**	***P*** **-value**
Age (years)	51.60 (14.08)	44.10 (6.11)	49.24 (6.46)^a^	50.77 (7.29)^a^	61.62 (9.88)^a,b,c^	<0.001
Male	2,927 (59.7%)	651 (42.1%)	268 (67.3%)^a^	761 (56.5%)^a,b^	1,247 (77.5%)^a,b,c^	<0.001
Smoker	1,609 (32.8%)	393 (25.4%)	173 (43.5%)^a^	440 (32.6%)^a,b^	603 (37.5%)^a,b^	<0.001
Alcohol drinker	1,705 (34.8%)	392 (25.5%)	160 (40.2%)^a^	476 (35.3%)^a^	677 (42.1%)^a,c^	<0.001
Systolic BP (mmHg)	130.72 ± 20.02	117.36 ± 13.72	124.24 ± 15.70^a^	134.16 ± 17.44^a,b^	142.26 ± 20.01^a,b,c^	<0.001
Diastolic BP (mmHg)	83.00 ± 11.07	78.31 ± 9.34	81.89 ± 10.31^a^	86.24 ± 10.67^a,b^	85.04 ± 11.55^a,b,c^	<0.001
FBG (mmol/L)	5.57 ± 1.49	5.19 ± 0.89	5.37 ± 1.02^a^	5.63 ± 1.51^a,b^	5.92 ± 1.89^a,b,c^	<0.001
LDL-C (mmol/L)	2.63 ± 0.77	2.52 ± 0.65	2.62 ± 0.71^a^	2.65 ± 0.80^a^	2.72 ± 0.84^a,b^	<0.001
HDL-C (mmol/L)	1.63 ± 0.45	1.69 ± 0.48	1.61 ± 0.40^a^	1.61 ± 0.43^a^	1.60 ± 0.44^a^	0.073
Total cholesterol (mmol/L)	5.04 ± 1.00	4.84 ± 0.86	5.08 ± 0.95^a^	5.02 ± 0.99^a^	5.23 ± 1.11^a,b,c^	<0.001
Body mass index (kg/m^2^)	24.92 ± 3.24	24.56 ± 3.12	24.88 ± 2.98^a^	25.28 ± 3.37^a^	24.96 ± 3.27^a,b,c^	0.006
Taking antihypertensive drug	1,182 (24.1%)	90 (5.8%)	56 (14.1%)^a^	369 (27.4%)^a,b^	667 (41.5%)^a,b,c^	<0.001
Taking hypoglycemic drug	258 (5.3%)	18 (1.2%)	13 (3.3%)^a^	77 (5.7%)^a^	177 (11.0%)^a,b,c^	<0.001

*Data are presented as mean ± standard deviation or n (%). baPWV, brachial-ankle pulse wave velocity; BP, blood pressure; FBG, fasting blood glucose; HDL-C, high-density lipoprotein-cholesterol; LDL-C, low-density lipoprotein-cholesterol*.

*Continuous variables were compared between groups using analysis of variance (ANOVA) and between-group post hoc analysis using Fisher's LSD if significant differences were revealed*.

*The P-values are for comparisons among the four subgroups*.

a *P < 0.05 vs. group 1 (no carotid plaque, normal baPWV)*;

b *P < 0.05 vs. group 2 (carotid plaque, normal baPWV)*;

c *P < 0.05 vs. group 3 (no carotid plaque, higher baPWV)*.

Participants were divided into four groups according to binary variables of carotid plaque and baPWV. The patients in the group with carotid plaque alone (n=398, 8.1%) were older, were predominantly males, smokers, alcohol drinkers, had been using antihypertensive and hypoglycemic drugs, and had higher BMI, SBP, DBP, FBG, TC, and LDL-C than the group with normal baPWV and no carotid plaque (*n* = 1,545, 31.5%). Similarly, the patients in the group with high baPWV alone (*n* = 1,348, 27.5%) were older, were predominantly males, smokers, alcohol drinkers, had been using antihypertensive and hypoglycemic drugs, and had higher BMI, SBP, DBP, FBG, TC, and LDL-C than the group with normal baPWV and no carotid plaque. Furthermore, the patients in the group with both carotid plaque and high baPWV (*n* = 1,608, 32.8%) were older, were predominantly males, had been using antihypertensive and hypoglycemic drugs, and had higher SBP, FBG, TC, and LDL-C than the groups with normal baPWV and no carotid plaque. The baseline characteristics stratified according to age, SBP, DBP, FBG, LDL-C, HDL-C, TC, and BMI are shown in Supplementary Table 1.

### CVD Events and All-Cause Mortality

After a mean follow-up of 5.68 ± 0.66 years, CVD events were observed in 167 participants, including 40 cases of MI and 127 cases of cerebral ischemic stroke. The incidence of CVD was 4.94 per 1,000 person-years. There were 204 all-cause deaths during follow-up, and the all-cause mortality was 7.02 per 1,000 person-years (Table 2).

**Table 2:** Association of carotid plaque and brachial-ankle pulse wave velocity status with all-cause mortality and cardiovascular disease events.

	**No. of events/all**	**Age-and sex-adjusted** **(95% CI)**	***P*** **-value**	Multivariable adjusted*(95% CI)	***P*** **-value**
**All-cause mortality (n = 4,899, deaths = 204)**					
No carotid plaque, normal baPWV	17/1,545	1.00 (reference)		1.00 (reference)	
Carotid plaque, normal baPWV	11/398	1.61 (0.75–3.46)	0.227	1.55 (0.71–3.45)	0.277
No carotid plaque, higher baPWV	26/1,348	1.04 (0.55–1.94)	0.906	0.84 (0.43–1.66)	0.616
Carotid plaque, higher baPWV	150/1,608	2.18 (1.21–3.91)	0.009	1.71 (0.90–3.26)	0.099
**Cardiovascular disease event**^**§**^**(*****n*** **= 4,899, events = 167)**					
No carotid plaque, normal baPWV	8/1,545	1.00 (reference)		1.00 (reference)	
Carotid plaque, normal baPWV	6/398	1.61 (0.49–5.24)	0.432	1.27 (0.38–4.28)	0.555
No carotid plaque, higher baPWV	44/1,348	4.15 (1.98–8.70)	0.001	2.68 (1.20–6.00)	0.007
Carotid plaque, higher baPWV	109/1,608	9.26 (4.45–19.29)	0.001	4.65 (2.06–10.45)	<0.001
**Cerebral ischemia (*****n*** **= 4,899, events = 127)**					
No carotid plaque, normal baPWV	4/1,545	1.00 (reference)		1.00 (reference)	
Carotid plaque, normal baPWV	2/398	1.73 (0.31–9.49)	0.526	1.51 (0.24–9.13)	0.655
No carotid plaque, higher baPWV	32/1,348	8.39 (2.95–23.84)	0.001	5.92 (1.76–19.93)	0.004
Carotid plaque, higher baPWV	89/1,608	16.84 (5.94–47.74)	<0.001	9.21 (2.71–31.19)	<0.001
**Myocardial infarction (*****n*** **= 4,899, events = 40)**					
No carotid plaque, normal baPWV	4/1,545	1.00 (reference)		1.00 (reference)	
Carotid plaque, normal baPWV	4/398	3.26 (0.80–13.25)	0.098	2.16 (0.62–7.53)	0.228
No carotid plaque, higher baPWV	12/1,348	3.09 (0.98–9.71)	0.053	1.93 (0.58–6.38)	0.279
Carotid plaque, higher baPWV	20/1,608	3.74 (1.14–12.28)	0.029	2.79 (0.68–11.49)	0.155

§ *Cerebral ischemia or myocardial infarction. Models 1 was adjusted for sex and age. Model 2 was adjusted for age, sex, smoking status, alcohol consumption, body mass index, fasting blood glucose, total cholesterol, low-density lipoprotein-cholesterol, high-density lipoprotein-cholesterol, systolic blood pressure, diastolic blood pressure, antihypertensive drug use, and hypoglycemic drug use. baPWV, brachial-ankle pulse wave velocity; CI, confidence interval; HR, hazard ratio*.

### Multivariable Cox Regression Analysis of Factors Associated With Adverse Outcomes

The Cox proportional hazards regression analyses are shown in Table 2. Carotid plaque alone (i.e., with normal baPWV) was not significantly associated with CVD events, cerebral infarction, MI, or all-cause mortality after adjustment for age and sex (model 1) or after adjustment for age, sex, smoking status, alcohol consumption, BMI, FBG, TC, LDL-C, HDL-C, SBP, DBP, antihypertensive drug use, and hypoglycemic drug use (model 2). Furthermore, in model 2, high baPWV alone was significantly associated with higher risks of CVD events (HR: 2.68; 95% CI: 1.20–6.00; *P* = 0.007) and cerebral infarction (HR: 5.92; 95% CI: 1.76–19.93; *P* = 0.004) but not MI or all-cause death. In addition, in model 2, the presence of both carotid plaque and high baPWV was associated with increased risks of CVD events (HR: 4.65; 95% CI: 2.06–10.45; *P* < 0.001) and cerebral infarction (HR: 9.21; 95% CI: 2.71–31.19; *P* < 0.001) but not MI or all-cause death. The C-index for CVD and all-cause death prediction were 0.669 (95% CI, 0.641–0.697) and 0.671 (95% CI, 0.643–0.699). For CVD, after the inclusion of carotid plaque, the proportion of correct classification increased by 60.1% (NRI = 0.601, Z = 7.111, *P* < 0.001), and the predictive power of model 2 was improved (IDI = 0.006, *P* < 0.001). For cerebral infarction, after the inclusion of carotid plaque, the proportion of correct classification increased by 59.7% (NRI = 0.597, Z = 6.988, *P* < 0.001), and the predictive power of model 2 was improved (IDI = 0.006, *P* < 0.001; Supplementary Table 2).

The results of the multivariable analysis of factors (model 2) associated with the rates of incident events are shown in Supplementary Figure 1. Age ≥ 50 years, current smoking, DBP ≥ 110 mmHg, antihypertensive drug therapy, high baPWV alone, and the combination of higher baPWV and carotid plaque were associated with cerebral infarction (*P* < 0.05 for all associations). Current smoking, DBP ≥ 110 mmHg, DBP <85 mmHg, FBG ≥ 7 mmol/L, antihypertensive drug therapy (borderline result), high baPWV alone, and the combination of high baPWV and carotid plaque were associated with CVD (*P* < 0.05 for all associations, except for *P* = 0.051 for the association of antihypertensive drug therapy with CVD events).

### Multivariable Cox Regression Analysis of Factors Associated With Adverse Outcomes in Subgroups Stratified by Age

Among participants ≥50 years old, high baPWV alone was associated with an increased risk of CVD events (HR: 4.52; 95% CI: 1.05–19.41; *P* = 0.042), but not with other outcomes (Table 3). For subjects aged ≥50 years old, the presence of both carotid plaque and high baPWV was associated with higher risks of CVD events (HR: 3.56; 95% CI: 1.25–10.51; *P* = 0.017) and cerebral infarction (HR: 5.68; 95% CI: 1.33–24.26; *P* = 0.019) but not MI or all-cause death (Table 3). Among participants aged 40–49 years old, the presence of both carotid plaque and high baPWV was associated with substantially higher risks of CVD events (HR: 12.33; 95% CI: 3.38–44.92; *P* < 0.001) and cerebral infarction (HR: 39.02; 95% CI: 54.25–358.12; *P* = 0.001), but not with MI or all-cause death (Table 3).

**Table 3:** Association of carotid plaque and brachial-ankle pulse wave velocity status with all-cause mortality and cardiovascular disease events in subgroups stratified according to age^*^.

	**40–49 years (*****n*** **= 2,184)**		**≥50 years (*****n*** **= 2,715)**	
	**HR (95% CI)**	***P*** **-value**	**HR (95% CI)**	***P*** **-value**
**All-cause mortality**				
No carotid plaque, normal baPWV	1.00 (reference)		1.00 (reference)	
Carotid plaque, normal baPWV	2.98 (0.67–13.27)	0.149	1.01 (0.39–2.60)	0.975
No carotid plaque, higher baPWV	1.13 (0.28–4.35)	0.862	0.53 (0.23–1.19)	0.122
Carotid plaque, higher baPWV	3.23 (0.79–13.26)	0.104	1.02 (0.49–2.14)	0.945
**Cardiovascular disease events** ^**§**^				
No carotid plaque, normal baPWV	1.00 (reference)		1.00 (reference)	
Carotid plaque, normal baPWV	0.00 (0.00–0.00)	0.990	1.41 (0.35–5.66)	0.629
No carotid plaque, higher baPWV	3.08 (0.87–10.91)	0.081	2.40 (0.81–7.04)	0.110
Carotid plaque, higher baPWV	12.33 (3.38–44.92)	<0.001	3.56 (1.25–10.51)	0.017
**Cerebral ischemia**				
No carotid plaque, normal baPWV	1.00 (reference)		1.00 (reference)	
Carotid plaque, normal baPWV	0.00 (0.00–0.00)	0.990	1.38 (0.19–9.85)	0.747
No carotid plaque, higher baPWV	8.22 (0.91–74.34)	0.061	4.52 (1.05–19.41)	0.042
Carotid plaque, higher baPWV	39.02 (4.25–358.12)	0.001	5.68 (1.33–24.26)	0.019
**Myocardial infarction**				
No carotid plaque, normal baPWV	1.00 (reference)		1.00 (reference)	
Carotid plaque, normal baPWV	0.00 (0.00–0.00)	0.991	2.82 (0.50–15.75)	0.237
No carotid plaque, higher baPWV	2.84 (0.53–15.46)	0.226	0.88 (0.15–5.10)	0.887
Carotid plaque, higher baPWV	1.51 (0.19–11.98)	0.694	1.35 (0.25–7.12)	0.721

* *The models were adjusted for age, sex, smoking status, alcohol consumption, body mass index, fasting blood glucose, total cholesterol, low-density lipoprotein-cholesterol, high-density lipoprotein-cholesterol, systolic blood pressure, diastolic blood pressure, antihypertensive drug use, and hypoglycemic drug use. baPWV, brachial-ankle pulse wave velocity; CI, confidence interval; HR, hazard ratio*.

§ *Cerebral ischemia or myocardial infarction*.

### Survival Analysis

**Figure 2:**
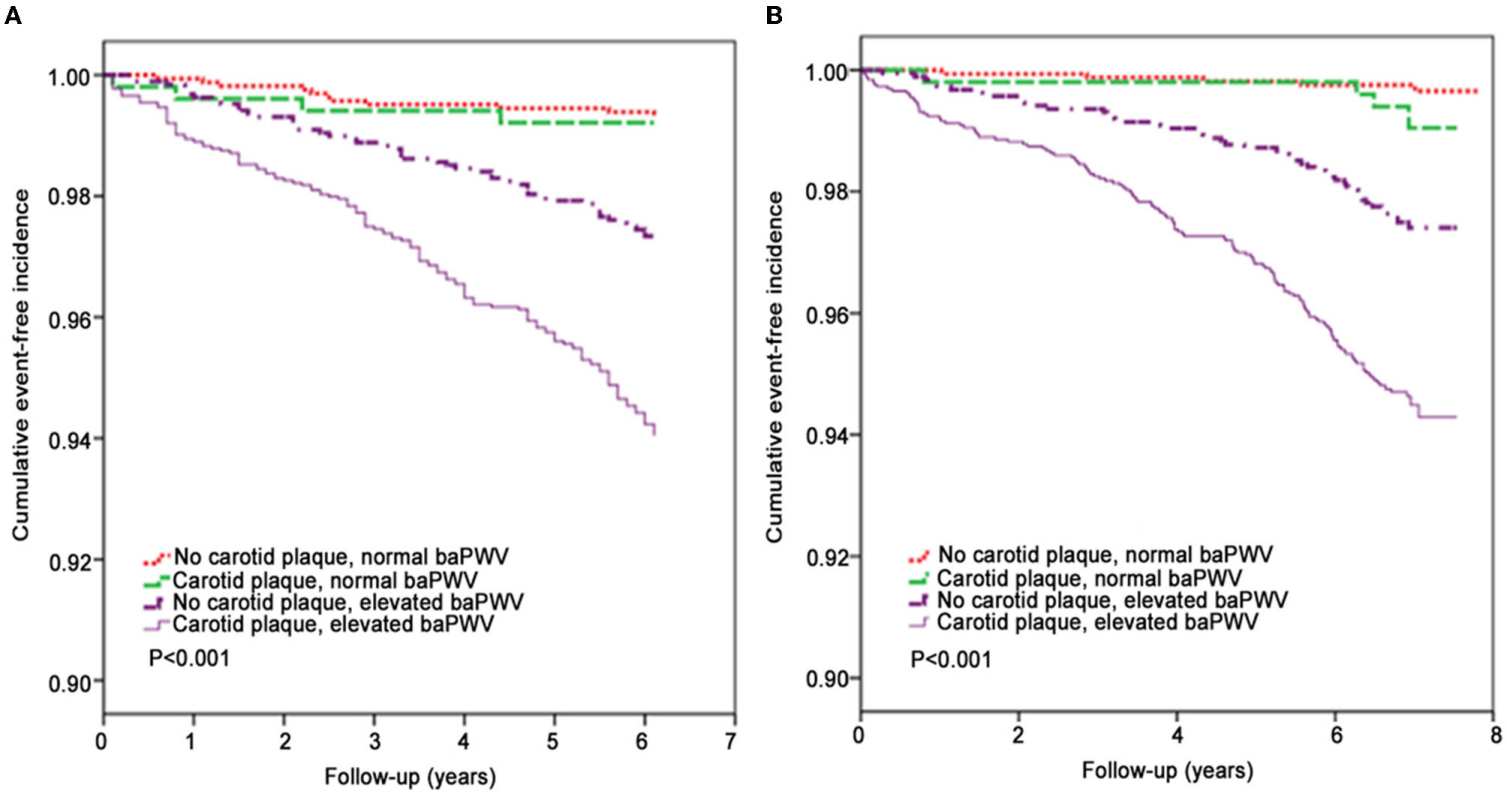
Kaplan-Meier analysis of the effects of carotid plaque and brachial-ankle pulse wave velocity on clinical outcomes. **(A)** Cardiovascular disease events (CVD, a composite of myocardial infarction and cerebral ischemic stroke). **(B)** Cerebral ischemic stroke events.

Kaplan-Meier analyses of the incidences of CVD events and cerebral infarction events revealed significant differences between groups (Figure 2). Notably, the cumulative incidences of CVD events and cerebral infarction were higher in participants with a combination of carotid plaque and higher baPWV than in the other groups (*P* < 0.001).

## Discussion

The present study showed that high baPWV alone was associated with increased risks of CVD events and cerebral infarction but not MI or all-cause death, whereas carotid plaque alone was not associated with increased risk of any of these adverse outcomes. More importantly, the presence of both carotid plaque and high baPWV was associated with a higher risk of CVD events and cerebral infarction compared with each factor alone, but not with increased risk of all-cause death. These results suggest that the presence of carotid atherosclerotic plaque and increased arterial stiffness might increase CVD risk, especially cerebral ischemic stroke.

Higher arterial stiffness and atherosclerotic carotid disease are known risk factors for CVD (4–8, 19–22); yet, the prediction value of both factors for CVD risk stratification has not been reported. In this study, the presence of both carotid artery atherosclerosis and increased arterial stiffness was seen in 32.8% of cases, while 8.1% of participants had carotid plaque alone and 27.5% of participants had high arterial stiffness alone. More importantly, we discovered that individuals with both carotid atherosclerosis and high baPWV had a higher risk of developing CVD events compared to those with carotid atherosclerosis or high arterial stiffness alone. Carotid plaque and high baPWV were associated with a 4.65-fold increased risk of CVD events (a composite of MI or cerebral infarction) and a 9.21-fold increased risk of ischemic cerebral infarction. The C-indexes were between 0.6 and 0.7, indicating fair concordance. These findings indicate a complex interaction between these two factors and their effect on CVD risk, especially cerebral infarction. The results also suggest that, in addition to traditional risk factors, screening for changes in vascular structure (atherosclerotic plaques) and function (arterial stiffness) might improve the accuracy of CVD risk assessment in high-risk groups. The J-BAVEL studies confirmed that the inclusion of ankle-brachial pressure index (an indicator of peripheral vascular occlusion) and baPWV (an indicator of arterial stiffness) might improve the accuracy of cardiovascular risk prediction by the Framingham risk model (37). Since carotid-femoral PWV (cfPWV) (38) is considered the “gold-standard” measure of arterial stiffness, future studies are warranted to further explore the interactions between cfPWV and carotid plaque and their association with the CVD risk.

This study showed that people with carotid atherosclerosis and high arterial stiffness had an increased risk of CVD than individuals with either of these factors alone. Sclerosis testing to evaluate target organ damage in patients with hypertension has been recommended by European (39), American (40), and Chinese guidelines (28). Still, due to the associated costs and limitations in equipment availability, the measurement of arterial stiffness is not commonly used in clinical practice. We recommend routine baPWV testing for middle-aged and elderly patients at first diagnosis.

The incidence of ischemic stroke is three times the incidence of ischemic heart disease in the Chinese population and is higher than in Western populations (41). In this study, we found that cerebral infarction risk increased 9.21-fold in participants with both baPWV and carotid plaque, which is particularly useful when screening Chinese population at risk for cerebral infarction.

It has been reported that statins can delay or even reverse subclinical vascular disease (42). Furthermore, a meta-analysis reported that each 1-mmol/L decrease in LDL-C is associated with a 21.1% reduction in the relative risk for stroke (43). Therefore, it would seem reasonable to recommend statins to those with high baPWV and carotid plaque to reduce LDL-C to 70 mg/dl or lower (44). Currently, there are no effective interventions to slow or reverse arterial stiffness. Studies have found that aerobic exercise (45), limiting sodium intake (46), and a Mediterranean diet (47) can prevent the progression of arteriosclerosis; hence people with high baPWV and carotid plaques should be encouraged to adopt a healthier lifestyle. In addition, previous studies confirmed that statin therapy could delay or even reverse subclinical vascular lesions, and patients with high baPWV combined with carotid plaque should take statins to reduce LDL-C below the 1.8-mmol/L target (44). In addition, a healthier lifestyle such as aerobic exercise (45) and a Mediterranean diet (48) could also help prevent atherosclerosis, thus effectively reducing the risk of cardiac CVD.

The above observations suggest that there might be some differences between the risk factors and pathological mechanisms underlying the development of carotid plaque (which reflects atherosclerotic disease in the carotid arteries) and increased baPWV (which reflects arteriosclerosis in peripheral arteries). In addition, the clinical repercussions also differ since arterial stiffness can lead to an acceleration of forward blood flow and an increase in the pulse wave, whereas carotid atherosclerosis leads to vascular occlusion and a decrease in blood flow. Nevertheless, although participants with high baPWV and carotid plaques had a significantly increased risk of CVD, this observational study was not designed to elucidate the underlying mechanisms. The possible mechanisms might include the following: (1) arteriosclerosis causes high-speed forward-flow damage to low-resistance organs and increases cardiac work and oxygen consumption and inadequate perfusion pressure in low-resistance organs during diastole (49, 50); (2) atherosclerosis causes lumen stenosis and reduces blood supply to target organs like the heart and brain; (3) those with steno-stiffness might be more susceptible to cardiovascular risk factors or may be exposed to more serious cardiovascular risk factors for longer periods of time; (4) carotid atherosclerosis and arterial stiffness have common risk factors that might cause CVD events through the steno-stiffness pathway and other pathways such as inflammation (51–53). Further research is needed to determine how carotid atherosclerosis and arterial stiffness interact to increase CVD risk.

Although this study population consisted of a primary prevention cohort, peripheral artery disease, which is a robust predictor of CVD outcomes (54), may influence the accurate measure of arterial stiffness. Otsuka et al. (54) reported that the ankle-brachial index (ABI) indicates peripheral artery embolism, which might affect the measurement of arterial hardness and increase the risk of cardiovascular diseases. Also, a previous study showed that patients > 60 years with carotid plaques and low ABI (ABI 0.9) have a significantly increased risk of ischemic cardiovascular disease (HR = 7.16) (55). The reduced risk of ABI is related to the increase of age. In the present study, the average age of patients was 51.6 ± 14.1 years, so the patients with abnormal ABI were not excluded, which is a limitation of this study. Nevertheless, this study has a large sample size, thus making the results still reliable. baPWV is largely affected by blood pressure. The cardiovascular arterial vascular Index (CAVI) does not depend on blood pressure parameters and may more objectively reflect the stiffness of arterial vessels, which is more conducive to the risk stratification assessment of CVD (56). We will further explore the predictive value of CAVI and CVD in future studies.

This study has a few limitations. First, the presence/absence of carotid plaque was treated as a binary variable, but the plaque characteristics were not considered. Subsequent studies are needed to evaluate the effects of plaque properties (such as area, thickness, and stenosis percentage) on CVD risk. Second, even though this study used baPWV to measure arterial stiffness, cfPWV may be a better choice. Still, baPWV is correlated with cfPWV (38), and the American Heart Association has included baPWV as a recommended indicator for arterial stiffness evaluation (57). Third, the follow-up was relatively short (5.68 ± 0.66 years) since CVD is a condition with a progression over many years, and CVD might have developed later in some participants. Fourth, the participants were workers in coal mines located in northern China and might not represent the general population in China.

## Conclusion

The presence of carotid plaque and high baPWV was associated with a high risk of CVD events and ischemic stroke. Moreover, the combination of carotid artery ultrasonography and baPWV measurement could predict the risk for CVD ability more accurately than a single measurement alone. Also, screening individuals for carotid plaque and arterial stiffness might help improve risk stratification and identify individuals in need of interventions to reduce CVD risk, especially cerebral ischemic stroke.

## Data Availability Statement

The original contributions presented in the study are included in the article/Supplementary Material, further inquiries can be directed to the corresponding author.

## Ethics Statement

The studies involving human participants were reviewed and approved by Kailuan General Hospital (Tangshan, China) and Beijing Tiantan Hospital. The patients/participants provided their written informed consent to participate in this study.

## Author Contributions

WL and YW carried out the studies, participated in collecting data, and drafted the manuscript. SC, JZ, and YF participated in the acquisition, data analysis, and statistical analysis. SW, JL, and JH participated in the study design. QS helped on revising the manuscript language. All authors read and approved the final manuscript.

## Funding

This study was supported by the National Key R&D Program (2020YFC2004703).

## Conflict of Interest

The authors declare that the research was conducted in the absence of any commercial or financial relationships that could be construed as a potential conflict of interest.

## Publisher's Note

All claims expressed in this article are solely those of the authors and do not necessarily represent those of their affiliated organizations, or those of the publisher, the editors and the reviewers. Any product that may be evaluated in this article, or claim that may be made by its manufacturer, is not guaranteed or endorsed by the publisher.

## Supplementary Material

Supplementary Figure 1 Multivariable analysis of the associations between baseline stratified variables and rates of incident events. There are separate multivariable models (model 2) for the incidence rates of cerebral infarction (CI) events and cardiovascular disease (CVD) events, respectively.

Supplementary Table 1 Stratified analysis of the baseline characteristics of the study population.

Supplementary Table 2 C-indexes of higher baPWV for f CVD events and cerebral infarction.
