# Supplementary figures and images for: Evaluation of Carotid Artery Atherosclerosis and Arterial Stiffness in Cardiovascular Disease Risk: An Ongoing Prospective Study From the Kailuan Cohort
Source: Front Cardiovasc Med. 2022 May 2;9:812652. doi: 10.3389/fcvm.2022.812652 (PMC9108697; doi:10.3389/fcvm.2022.812652)

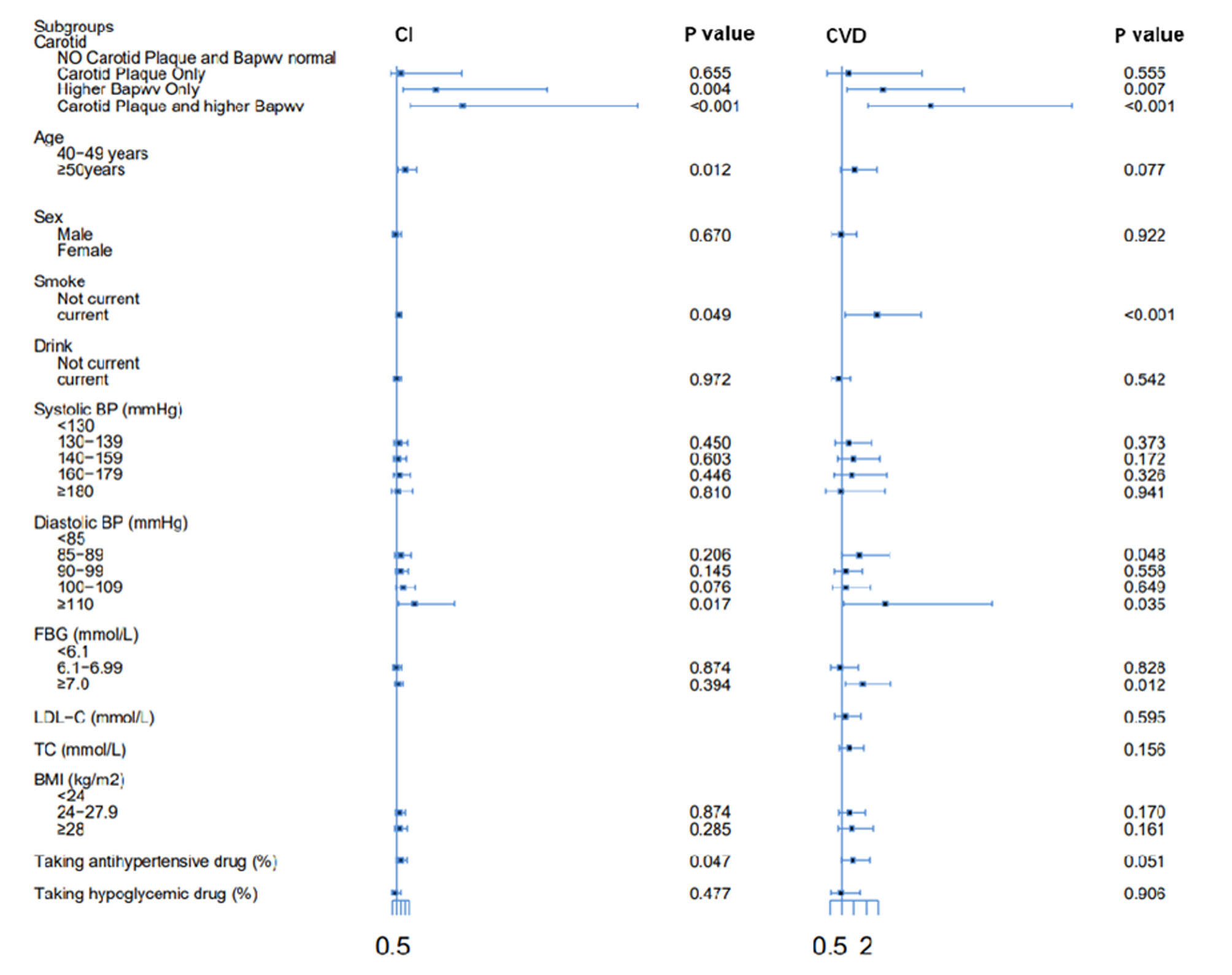

Supplement: Supplementary Figure 1 — Multivariable analysis of the associations between baseline stratified variables and rates of incident events. There are separate multivariable models (model 2) for the incidence rates of cerebral infarction (CI) events and cardiovascular disease (CVD) events, respectively. [file Image_1.TIF]
