# Supplementary material for: Evaluation of Carotid Artery Atherosclerosis and Arterial Stiffness in Cardiovascular Disease Risk: An Ongoing Prospective Study From the Kailuan Cohort
Source: Front Cardiovasc Med. 2022 May 2;9:812652. doi: 10.3389/fcvm.2022.812652 (PMC9108697; doi:10.3389/fcvm.2022.812652)
Supplement: Supplementary Table 1 — Stratified analysis of the baseline characteristics of the study population. [file Table_1.doc]

**Supplementary Table 1.** Stratified analysis of the baseline characteristics of the study populations

|  | No carotid plaque, normal baPWV  (*n* = 1545) | Carotid plaque, normal baPWV  (*n* = 398) | No carotid plaque, higher baPWV  (*n* = 1348) | Carotid plaque,  higher baPWV  (*n* = 1608) | All participants  (*n* = 4899) | *P*-value |
| --- | --- | --- | --- | --- | --- | --- |
| Age |  |  |  |  |  |  |
| 40–49 years, *n* (%) | 1131 (73.2%) | 178 (44.7%) | 624 (46.3%) | 251 (15.6%) | 2184 (44.6%) | <0.001 |
| ≥50 years, *n* (%) | 414 (26.8%) | 220 (55.3%) | 724 (53.7%) | 1357 (84.4%) | 2715 (55.4%) | <0.001 |
| 40–49 years, mean ± SD | 44.29 ± 2.66 | 45.27 ± 2.63 | 44.99 ± 2.74 | 45.97 ± 2.64 | 45.13 ± 2.68 | <0.001 |
| ≥50 years, mean ± SD | 58.69 ± 3.58 | 61.02 ± 4.73 | 64.44 ± 3.64 | 65.25 ± 4.82 | 62.28 ± 4.19 | <0.001 |
| All, mean ± SD | 46.79 ± 5.30 | 51.66 ± 7.90 | 52.28 ± 8.99 | 63.34 ± 12.47 | 54.18 ± 11.52 | <0.001 |
| Male, *n* (%) | 651 (42.1%) | 268 (67.3%) | 761 (56.5%) | 1247 (77.5%) | 2927 (59.7%) | <0.001 |
| Smoker, *n* (%) | 393 (25.4%) | 173 (43.5%) | 440 (32.6%) | 603 (37.5%) | 1609 (32.8%) | <0.001 |
| Alcohol drinker, *n* (%) | 392 (25.4%) | 160 (40.2%) | 476 (35.3%) | 677 (42.1%) | 1705 (34.8%) | <0.001 |
| Systolic blood pressure |  |  |  |  |  |  |
| <130 mmHg, *n* (%) | 1197 (77.5%) | 227 (57.0%) | 489 (36.3%) | 375 (23.3%) | 2288 (46.7%) | <0.001 |
| 130–139 mmHg, *n* (%) | 234 (15.1%) | 103 (25.9%) | 362 (26.8%) | 331 (20.6%) | 1030 (21.0%) | <0.001 |
| 140–159 mmHg, *n* (%) | 105 (6.8%) | 58 (14.6%) | 373 (27.7%) | 557 (34.7%) | 1093 (22.3%) | <0.001 |
| 160–179 mmHg, *n* (%) | 9 (0.6%) | 8 (2.0%) | 96 (7.1%) | 267 (16.6%) | 380 (7.8%) | <0.001 |
| ≥180 mmHg, *n* (%) | 0 (0%) | 2 (0.5%) | 28 (2.1%) | 78 (4.8%) | 108 (2.2%) | <0.001 |
| <130 mmHg, mean ± SD | 111.98 ± 9.74 | 113.29 ± 8.81 | 117.53 ± 7.66 | 117.91 ± 7.51 | 115.11 ± 8.43 | <0.001 |
| 130–139 mmHg, mean ± SD | 131.62 ± 2.81 | 132.67 ± 3.56 | 132.30 ± 3.19 | 132.32 ± 3.21 | 132.22 ± 3.19 | 0.014 |
| 140–159 mmHg, mean ± SD | 143.36 ± 4.67 | 144.56 ± 5.11 | 145.48 ± 5.68 | 146.63 ± 5.85 | 145.21 ± 5.33 | <0.001 |
| 160–179 mmHg, mean ± SD | 162.77 ± 6.14 | 164.04 ± 4.18 | 165.08 ± 6.23 | 166.10 ± 6.25 | 164.49 ± 5.70 | <0.001 |
| ≥180 mmHg, mean ± SD | 0 | 180.00 ± 0.00 | 188.45 ± 10.91 | 188.00 ± 10.85 | 185.48 ± 10.89 | 0.201 |
| All, mean ± SD | 117.36 ± 13.72 | 124.24 ± 15.70 | 134.16 ± 17.44 | 142.26 ± 20.01 | 130.72 ± 20.02 | <0.001 |
| Diastolic blood pressure |  |  |  |  |  |  |
| <85 mmHg, *n* (%) | 1222 (79.1%) | 258 (64.8%) | 665 (49.3%) | 832 (51.7%) | 2977 (60.8%) | <0.001 |
| 85–89 mmHg, *n* (%) | 111 (7.2%) | 37 (9.3%) | 140 (10.4%) | 140 (8.7%) | 428 (8.7%) | <0.001 |
| 90–99 mmHg, *n* (%) | 169 (10.9%) | 71 (17.8%) | 345 (25.6%) | 413 (25.7%) | 998 (20.4%) | <0.001 |
| 100–109 mmHg, *n* (%) | 39 (2.5%) | 29 (7.3%) | 159 (11.8%) | 192 (11.9%) | 419 (8.5%) | <0.001 |
| ≥110 mmHg, *n* (%) | 4 (0.3%) | 3 (0.8%) | 39 (2.9%) | 31 (2.0%) | 77 (1.6%) | <0.001 |
| <85 mmHg, mean ± SD | 74.76 ± 6.62 | 75.82 ± 5.94 | 77.54 ± 5.18 | 76.03 ± 6.64 | 76.04 ± 6.10 | <0.001 |
| 85–89 mmHg, mean ± SD | 87.53 ± 1.31 | 87.36 ± 1.37 | 87.43 ± 1.25 | 87.72 ± 1.35 | 87.51 ± 1.32 | 0.225 |
| 90–99 mmHg, mean ± SD | 91.64 ± 2.67 | 92.23 ± 3.12 | 92.25 ± 3.12 | 92.21 ± 3.03 | 92.08 ± 2.99 | 0.150 |
| 100–109 mmHg, mean ± SD | 101.23 ± 2.30 | 101.21 ± 2.57 | 101.31 ± 2.53 | 101.71 ± 2.84 | 101.36 ± 2.56 | 0.456 |
| ≥110 mmHg, mean ± SD | 110.66 ± 4.76 | 114.00 ± 5.29 | 114.51 ± 4.45 | 115.17 ± 5.65 | 113.58 ± 5.01 | 0.394 |
| All, mean ± SD | 78.31 ± 9.34 | 81.89 ± 10.31 | 86.24 ± 10.67 | 85.04 ± 11.55 | 83.00 ± 11.07 | <0.001 |
| Fasting blood glucose |  |  |  |  |  |  |
| <6.1 mmol/L, *n* (%) | 1424 (92.1%) | 349 (87.7%) | 1097 (81.4%) | 1169 (72.7%) | 4039 (82.4%) | <0.001 |
| 6.1–6.99 mmol/L, *n* (%) | 79 (5.1%) | 28 (7.0%) | 132 (9.8%) | 183 (11.4%) | 422 (8.7%) | <0.001 |
| ≥7.0 mmol/L, *n* (%) | 42 (2.7%) | 21 (5.3%) | 119 (8.8%) | 256 (15.9%) | 438 (8.9%) | <0.001 |
| <6.1 mmol/L, mean ± SD | 5.01 ± 0.48 | 5.08 ± 0.50 | 5.12 ± 0.49 | 5.09 ± 0.49 | 5.08 ± 0.49 | <0.001 |
| 6.1–6.99 mmol/L, mean ± SD | 6.37 ± 0.22 | 6.40 ± 0.24 | 6.45 ± 0.27 | 6.46 ± 0.26 | 6.42 ± 0.25 | 0.042 |
| ≥7.0 mmol/L, mean ± SD | 8.91 ± 1.89 | 8.31 ± 1.68 | 9.30 ± 2.58 | 9.33 ± 2.56 | 8.96 ± 2.18 | 0.216 |
| All, mean ± SD | 5.19 ± 0.89 | 5.37 ± 1.02 | 5.63 ± 1.51 | 5.92 ± 1.89 | 5.57 ± 1.49 | <0.001 |
| Low-density lipoprotein cholesterol |  |  |  |  |  |  |
| <4.1 mmol/L, *n* (%) | 1410 (91.2%) | 356 (89.4%) | 1154 (85.6%) | 1330 (82.7%) | 4247 (86.7%) | <0.001 |
| ≥4.1 mmol/L, *n* (%) | 135 (8.8%) | 42 (10.6%) | 194 (14.4%) | 278 (17.3%) | 652 (13.3%) | <0.001 |
| <4.1 mmol/L, mean ± SD | 2.49 ± 0.60 | 2.54 ± 0.61 | 2.59 ± 0.67 | 2.62 ± 0.68 | 2.56 ± 0.64 | <0.001 |
| ≥4.1 mmol/L, mean ± SD | 4.61 ± 0.53 | 4.48 ± 0.37 | 4.95 ± 1.93 | 4.73 ± 1.25 | 4.69 ± 1.02 | <0.001 |
| All, mean ± SD | 2.52 ± 0.65 | 2.62 ± 0.71 | 2.65 ± 0.80 | 2.72 ± 0.84 | 2.63 ± 0.77 | <0.001 |
| High-density lipoprotein cholesterol |  |  |  |  |  |  |
| <1.0 mmol/L, *n* (%) | 31 (2.0%) | 12 (3.0%) | 63 (4.7%) | 86 (5.3%) | 192 (3.9%) | <0.001 |
| ≥1.0 mmol/L, *n* (%) | 1514 (98.0%) | 386 (97.0%) | 1285 (95.3%) | 1522 (94.7%) | 4707 (96.1%) | 0.247 |
| <1.0 mmol/L, mean ± SD | 0.88 ± 0.10 | 0.89 ± 0.07 | 0.87 ± 0.12 | 0.89 ± 0.09 | 0.88 ± 0.09 | 0.880 |
| ≥1.0 mmol/L, mean ± SD | 1.70 ± 0.47 | 1.63 ± 0.39 | 1.64 ± 0.41 | 1.63 ± 0.42 | 1.65 ± 0.42 | 0.165 |
| All, mean ± SD | 1.69 ± 0.48 | 1.61 ± 0.40 | 1.61 ± 0.43 | 1.60 ± 0.44 | 1.63 ± 0.45 | <0.001 |
| Total cholesterol |  |  |  |  |  |  |
| <6.2 mmol/L, *n* (%) | 1067 (69.1%) | 238 (59.7%) | 829 (61.5%) | 861 (53.5%) | 2995 (61.1%) | 0.456 |
| ≥6.2 mmol/L, *n* (%) | 478 (30.9%) | 160 (40.3%) | 519 (38.5%) | 747 (46.5%) | 1904 (38.9%) | <0.001 |
| <6.2 mmol/L, mean ± SD | 4.70 ± 0.70 | 4.81 ± 0.69 | 4.78 ± 0.70 | 4.88 ± 0.74 | 4.79 ± 0.71 | <0.001 |
| ≥6.2 mmol/L, mean ± SD | 6.74 ± 0.50 | 6.82 ± 0.51 | 6.89 ± 0.94 | 6.98 ± 0.95 | 6.85 ± 0.73 | <0.001 |
| All, mean ± SD | 4.84 ± 0.86 | 5.08 ± 0.95 | 5.02 ± 0.99 | 5.23 ± 1.11 | 5.04 ± 1.00 | <0.001 |
| Body mass index |  |  |  |  |  |  |
| <24 kg/m^2^, *n* (%) | 711 (46.0%) | 158 (39.7%) | 489 (36.3%) | 624 (38.8%) | 1982 (40.5%) | <0.001 |
| 24–27.9 kg/m^2^, *n* (%) | 637 (41.2%) | 185 (46.5%) | 601 (44.6%) | 715 (44.5%) | 2138 (43.6%) | <0.001 |
| ≥28 kg/m^2^, *n* (%) | 197 (12.8%) | 55 (13.8%) | 258 (19.1%) | 269 (16.7%) | 779 (15.9%) | <0.001 |
| <24 kg/m^2^, mean ± SD | 21.94 ± 1.36 | 22.06 ± 1.58 | 21.92 ± 1.62 | 21.78 ± 1.64 | 21.92 ± 1.55 | 0.104 |
| 24–27.9 kg/m^2^, mean ± SD | 25.72 ± 1.35 | 25.80 ± 1.10 | 25.89 ± 1.15 | 25.85 ± 1.18 | 25.82 ± 1.19 | 0.052 |
| ≥28 kg/m^2^, mean ± SD | 30.22 ± 2.04 | 29.78 ± 1.78 | 30.19 ± 2.23 | 29.96 ± 1.84 | 30.03 ± 1.97 | 0.299 |
| All, mean ± SD | 24.56 ± 3.12 | 24.88 ± 2.98 | 25.28 ± 3.37 | 24.96 ± 3.27 | 24.92 ± 3.24 | <0.001 |
| Taking antihypertensive drug (%) | 90 (5.8%) | 56 (14.1%) | 369 (27.4%) | 667 (41.5%) | 1182 (24.1%) | <0.001 |
| Taking hypoglycemic drug (%) | 18 (1.2%) | 13 (3.3%) | 77 (5.7%) | 177 (11.0%) | 258 (5.3%) | <0.001 |

baPWV: brachial-ankle pulse wave velocity; SD: standard deviation. The *P*-values are for comparisons among the four subgroups.
