# Supplementary material for: Evaluation of Carotid Artery Atherosclerosis and Arterial Stiffness in Cardiovascular Disease Risk: An Ongoing Prospective Study From the Kailuan Cohort
Source: Front Cardiovasc Med. 2022 May 2;9:812652. doi: 10.3389/fcvm.2022.812652 (PMC9108697; doi:10.3389/fcvm.2022.812652)
Supplement: Supplementary Table 2 — C-indexes of higher baPWV for f CVD events and cerebral infarction. [file Table_2.doc]

**Supplementary Table S2.** C-indexes of higher baPWV for f CVD events and cerebral infarction

|  | Model 1 (higher baPWV) | Model 2 (higher baPWV + carotid plaque) | P-value |
| --- | --- | --- | --- |
| CVD |  |  |  |
| C-index | 0.658 | 0.715 | <0.001 |
| Cerebral infarction |  |  |  |
| C-index | 0.647 | 0.700 | <0.001 |
